# Supplementary material for: Elevated RBP4 in Subclinical Ketosis Cows Inhibits Follicular Granulosa Cell Proliferation and Steroid Hormone Synthesis
Source: Animals (Basel). 2024 Oct 29;14(21):3118. doi: 10.3390/ani14213118 (PMC11545013; doi:10.3390/ani14213118)
Supplement: Supplementary file 1 [file animals-14-03118-s001.zip › animals-3168156-supplementary.pdf]

Supplemental Table S1

Nutritional composition of the diets used for early lactation in lactating Holstein cows.

Item(% of DM, unless noted)	Measurement
Diet composition	
Lucerne	6.25
Cottonseed	2.5
Oat skin	1.25
Soyabean meal	3.25
Lactobacillus	3
Corn	7.5
Molasses	2.5
Silage	63.5
NaCl	0.8
Premix	8.25
$NaHCO_3$	1.2
Total	100.0
Nutritional level %	
Net milk production(Mcal/kg)	0.78
Crude protein	17.7
Starch	22.7
Dry matter	48.0
Dry matter intake	23.5
Neutral detergent fibers	31.5
Acid detergent fibers	19.0
Roughage source neutral	18.7

Supplemental Table S2: Basic Information of Cows in SCK-AE and C-E Groups.

Index	C-E	SCK-AE
Headcount	12	12
Age (in years)	2.95±2.24	2.59±1.60
parity	2.09±1.75	1.92±1.32
14-21 d BCS (points)	3.07 ± 0.03	3.26 ± 0.04
45-60 d BCS (points)	2.83 ± 0.07	2.69 ± 0.06
Lactation (kg/d)	34.96 ± 3.93	34.39 ± 3.71
